# Bioactive adrenomedullin a prognostic biomarker in patients with mild to moderate dyspnea at the emergency department: an observational study

**DOI:** 10.1007/s11739-021-02776-y

**Published:** 2021-06-26

**Authors:** Kevin Bronton, Torgny Wessman, Klas Gränsbo, Janin Schulte, Oliver Hartmann, Olle Melander

**Affiliations:** 1 Department of Clinical Sciences, Lund University, Jan Waldenströms gata 35, 214 28 Malmö, Sweden; 2 Department of Internal Medicine, Skåne University Hospital, Malmö, Sweden; 3 Department of Emergency Medicine, Skåne University Hospital, Malmö, Sweden; 4 SphingoTec GmbH, Hennigsdorf, Brandenburg, Germany

**Keywords:** Bioactive adrenomedullin, Heart failure, Dyspnea, Emergency department, Prediction

## Abstract

Acute dyspnea with underlying congestion is a leading cause of emergency department (ED) visits with high rates of hospitalization. Adrenomedullin is a vasoactive neuropeptide hormone secreted by the endothelium that mediates vasodilation and maintains vascular integrity. Plasma levels of biologically active adrenomedullin (bio-ADM) predict septic shock and vasopressor need in critically ill patients and are associated with congestion in patients with acute heart failure (HF) but the prognostic value in unselected dyspneic patients at the ED is unknown. The purpose of this study is to test if bio-ADM predicts adverse outcomes when sampled in patients with acute dyspnea at presentation to the ED. In this single-center prospective observational study, we included 1402 patients from the ADYS (Acute DYSpnea at the Emergency Department) cohort in Malmö, Sweden. We fitted logistic regression models adjusted for sex, age, N-terminal pro-B-type natriuretic peptide (NT-proBNP), creatinine, and C-reactive protein (CRP) to associate bio-ADM plasma levels to mortality, hospitalization, intravenous (IV) diuretic treatment and HF diagnosis. Using receiver operating characteristic (ROC) curve analysis we evaluated bio-ADM discrimination for these outcomes compared to a reference model (sex, age, NT-proBNP, creatinine, and CRP). Model performance was compared by performing a likelihood ratio test on the deviances of the models. Bio-ADM (per interquartile range from median) predicts both 90-day mortality [odds ratio (OR): 1.5, 95% confidence interval (CI) 1.2−2.0, *p* < 0.002] and hospitalization (OR: 1.5, 95% CI 1.2−1.8, *p* < 0.001) independently of sex, age, NT-proBNP, creatinine, and CRP. Bio-ADM statistically significantly improves the reference model in predicting mortality (added *χ*^2^ 9.8, *p* = 0.002) and hospitalization (added *χ*^2^ 14.1, *p* = 0.0002), and is associated with IV diuretic treatment and HF diagnosis at discharge. Plasma levels of bio-ADM sampled at ED presentation in acutely dyspneic patients are independently associated with 90-day mortality, hospitalization and indicate the need for decongestive therapy.

## Introduction

Patients experiencing shortness of breath have been reported to account for close to one in ten visits to the emergency department (ED). Reports suggest that up to half of these patients get admitted for further in-hospital workup. The reasons behind such high admittance rates range from the fact that undifferentiated dyspnea may be caused by potentially life-threatening conditions while the diagnostic process is often complicated by the presence of several comorbidities, particularly among the elderly [1]. Congestive heart failure (CHF) is one of the main factors behind the burden of disease worldwide and of which dyspnea is a cardinal sign of exacerbation. In current practice, the ED physician has a limited supply of clinically useful diagnostic tools available to both the most effective treatment to initiate at the ED and decide whether in-patient management for further workup is required.

In the differential diagnostic workup for CHF, current practice entails a physical examination, vital signs, electrocardiogram, laboratory tests, and common imaging modalities [2]. Nevertheless, the prognostication and preliminary diagnosis of unselected acute dyspnea during the first hour at the ED, which determines the level of care and acute treatment, remains a huge challenge.

Adrenomedullin (ADM) is a neurohormonal peptide first discovered in pheochromocytoma-derived tissue in 1993. It is expressed in a multitude of cells and organ systems, and apart from adrenal medullae, the vascular endothelium is a significant site of secretion [3, 4]. Activation of the ADM precursor hormone (pre-proADM) starts with endopeptidase cleavage to an intermediate form of ADM (ADM-glycine). However, to become biologically active, the ADM-glycine needs to be amidated by the enzyme peptidyl ∝-amidating monooxygenase (PAM) to form the biologically active form of ADM, bioactive ADM (bio-ADM) [5]. The most well-established cardiovascular effects of bio-ADM are to mediate vasodilation, natriuresis, and maintain vascular integrity [2]. Once formed, circulating bio-ADM has a half-life of about 22 min and is actively degraded by proteases [6].

Whereas an inactive fragment of the ADM pre-pro-hormone (midregional pro-ADM) has been shown to add prognostic value regarding 90-day mortality in acute heart failure in addition to B-type natriuretic peptide (BNP) [7], a recently developed assay measuring bio-ADM opened up for studies more specifically quantifying the biological activity of the hormone in the circulation [8]. Recent studies have shown that circulating levels of bio-ADM correlate significantly with outcomes in both sepsis [9–15] and heart failure [2, 16–21], which have pathophysiological similarities in disrupted microcirculatory homeostasis [22, 23]. However, the prognostic value of bio-ADM in unselected patients with acute dyspnea, sampled at the immediate entry to the ED has not been studied.

We hypothesize that high levels of bio-ADM that indicate endothelial dysfunction among unselected acute dyspnea patients might identify individuals with severe illness including congestion, requiring a higher level of care and immediate decongestive therapy. To address this hypothesis, we set out to test whether plasma levels of bio-ADM, measured upon immediate admission to the ED, predict 90-day mortality and hospitalization (primary outcomes), as well as evaluating indirect measures of congestion such as the need of diuretics and diagnosed CHF among unselected patients with undifferentiated acute dyspnea.

## Materials and methods

### Collection of patient data

The study was conducted by analyzing the ADYS (Acute DYSpnea at the Emergency Department) cohort which contains clinical data and plasma samples from patients who presented to the ED of Skåne University Hospital in Malmö (SUS Malmö), and responsible for a catchment area of nearly 400,000 people and sees up to 85,000 visits per year. Patients 18 years of age and older who presented to the ED during daytime, 6:45 AM to 4:30 PM, with acute dyspnea between March 6th 2013 and April 11th, 2018 were approached by a research nurse and asked whether they wanted to enroll and be included in the ADYS cohort. Exclusion criteria included presenting unconscious or demanding intensive care. We obtained both written and oral consent from all participants and the study was approved by the Regional Ethical Review Board in Lund (Dnr 2012/762).

Participants were triaged according to Medical Emergency Triage and Treatment System-Adult score (METTS-A) and had their blood sampled. A total of 1710 patients were recruited to the cohort by April 11th, 2018. After inclusion, participants were questioned about past medical history and comorbidities and were asked to provide a list of current medications. The research nurses confirmed the details by reviewing the past medical history of the patients in the electronic health record and could request support from senior physicians whenever uncertainties occurred.

**Fig. 1:**
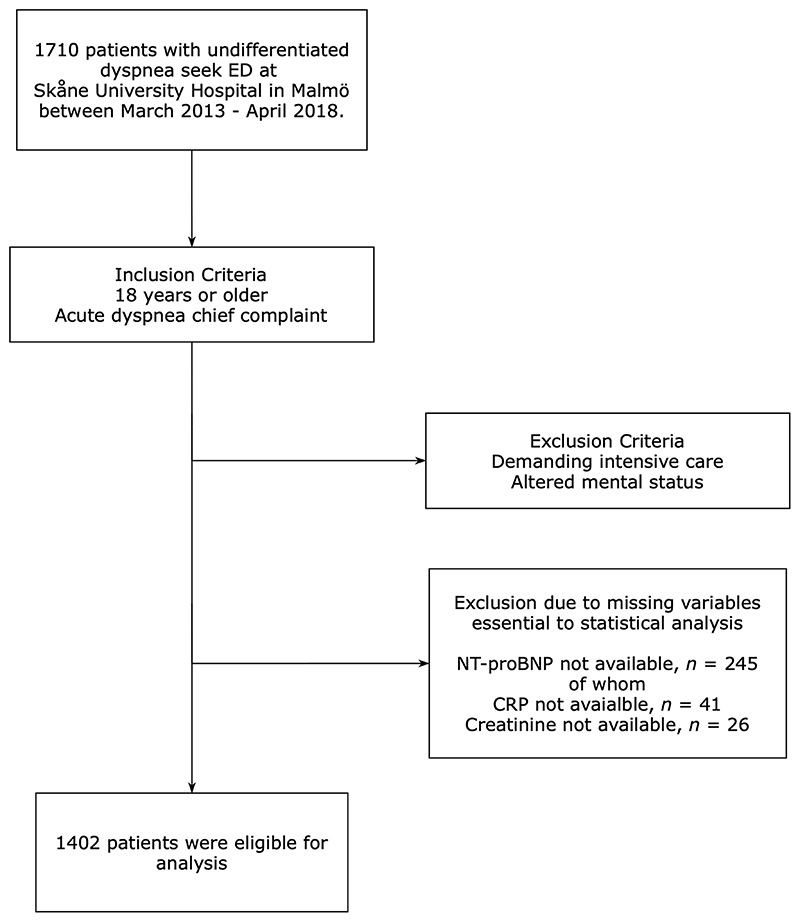
Flowchart of enrollment process from the ADYS-cohort (*n* = 1402)

In preparation for the statistical analyses of this study, we excluded 308 patients due to missing blood samples or other data essential to the statistical analyses and also, if they lacked a Swedish Personal identity number which prevents matching against the national civil registry. 1402 patients were eligible for the final statistical analysis. See Fig. 1 for the enrollment exclusion process.

Plasma samples were drawn within an hour of presentation to the ED and then frozen within 2 h of collection and stored at—80 °C until analysis. C-reactive protein (CRP) (Atellica CH930, Siemens Healthcare Diagnostics Inc) and serum creatinine (enzymatic colorimetric assay, Cobas NPU04998, Roche Diagnostics) were analyzed during the course of ED care by the local certified laboratory at the department of clinical chemistry of Skåne University Hospital. Bio-ADM was measured in duplicates using a one-step microtiter plate-based sandwich chemiluminescence immunoassay (SphingoTec GmbH, Hennigsdorf, Germany) at a laboratory located in Germany by professionals blinded to clinical data. N-terminal pro-B-type natriuretic peptide (NT-proBNP) was measured using a one-step automated sandwich electrochemiluminescence immunoassay (Cobas e411, Roche Diagnostics) in March 2020.

### Outcome definitions

The primary outcome measure was 90-day all-cause mortality. Mortality status was retrieved from the Swedish National Civil Register and hospitalization through review of the medical health records (MHR) of the subjects. As secondary outcomes, we specified shorter-term outcome measures analyzing both 7 and 30-day mortality, hospitalization, intravenous (IV) diuretic treatment given during the time at the ED, and CHF as a stated diagnosis at discharge, either from the ED or in-hospital ward. These were retrieved from reviewing the subject’s MHR. CHF diagnosis was made based on a local clinical aid document based on the ESC guidelines for acute and chronic heart failure [24]; outlining the presence of symptoms such as breathlessness, orthopnea, paroxysmal nocturnal dyspnea, reduced exercise tolerance, ankle swelling or sudden weight gain.

### Statistical analysis

Results are presented as number (n) and proportions as percentage (%), mean and standard deviation (SD), or median and interquartile range (IQR), depending on the distribution. Histograms were used to assess variables for normality. Monotonic trends across bio-ADM quartiles were evaluated with Cochran−Armitage test for categorical variables, Johnckheere−Terpstra test in non-normally distributed continuous variables, or Spearman rank correlation for trends in normally distributed continuous variables, as suitable. All biomarker data were log-transformed using the natural logarithm. Logistic regression models were fitted to analyze the predictive value of bio-ADM and other variables in both uni- and multivariate analyses. For continuous variables, odds ratio (OR) was standardized to describe the OR for change of one IQR from the median value of the log-transformed biomarker. Survival curves plotted by the Kaplan−Meier method using quartiles of bio-ADM were used for illustrative purposes. Receiver operating characteristic (ROC) curve analysis was used to evaluate predictive capacity for 90-day mortality and hospital admission. Optimal discrimination cutoff was determined by Youden’s J statistic [25]. Nested logistic regression model fit was compared by Likelihood Ratio Test, computing the difference in deviance for the specified models [26]. A two-sided *p* value of 0.05 was considered statistically significant. 95% confidence intervals (CI) are presented. The statistical analyses were performed using R version 3.6.3 (2020-02-29, “Holding the Windsock”, Copyright © 2020 The R Foundation for Statistical Computing).

## Results

### Baseline characteristics

Among the 1402 patients analyzed in the study 779 (56%) patients were female. The mean age was 71 (SD: ± 17) years. With regard to biomarkers in the studied cohort, median CRP was 10 (IQR: 3−35) mg/L, median serum creatinine was 80 (IQR: 65−106) μmol/L, median NT-proBNP was 869 (IQR: 153−3828) pg/mL, and lastly, median bio-ADM was 28 (IQR: 65−106) pg/mL.

Dividing the cohort into quartiles based on bio-ADM concentration the following was observed: Proportion of females decreased with increasing bio-ADM levels (*p* < 0.005). With respect to age, patients with lower bio-ADM levels were younger, but this trend seems to flatten above the median (*p*-trend < 0.005). With regard to prevalent disease, there was an increasing proportion of patients with CHF with higher bio-ADM levels (*p*-trend < 0.001). This is also true for the proportion of prevalent chronic obstructive pulmonary disease (COPD) (*p*-trend < 0.005) but not for undifferentiated infection at triage (*p*-trend > 0.9). Bio-ADM had a positive monotonic correlation with CRP, NT-proBNP, and serum creatinine (all *p*-trend < 0.005). Study population characteristics are summarized in Table 1.

### Primary outcomes: 90-day mortality

**Fig. 2:**
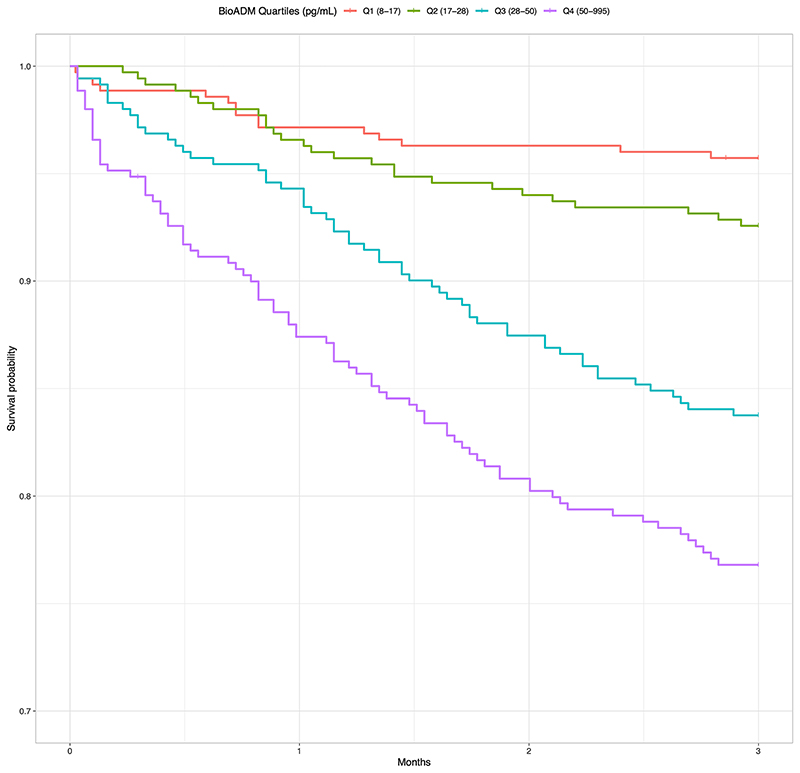
3 month (90 day) survival curves for the study population (*n* = 1402) stratified by quartiles of bioactive adrenomedullin (bio-ADM). Note cut Y-axis

Overall 179 (12.8%) patients died within 90 days of their ED visit. There was an ascending trend in 90-day mortality with increasing concentrations of bio-ADM. Dividing the study population into quartiles based on bio-ADM concentration, 90-day mortality was 4.3%, 7.4%, 16.2%, 23.1% per quartile in ascending order. Survival curves for each bio-ADM quartile are illustrated in Fig. 2.

**Fig. 3:**
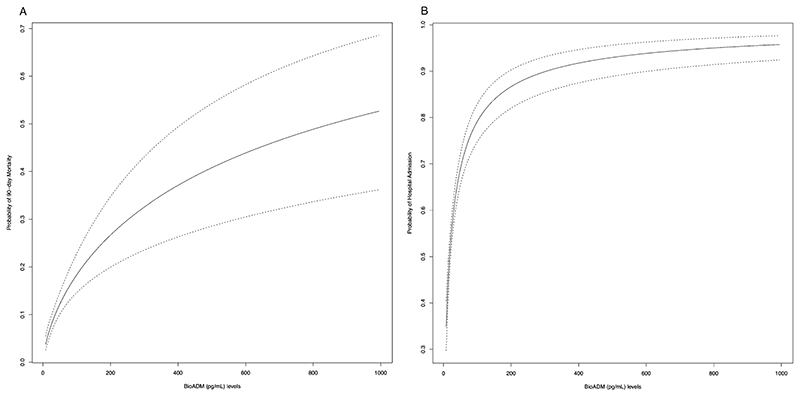
Functions of bioactive adrenomedullin (bio-ADM) and probability of a primary outcome event occurring (90 day all-cause mortality or hospitalization)

There was an increasing probability for both 90-day mortality and hospitalization with higher bio-ADM levels (Fig. 3). See Supplementary Figure S1 for change in log Odds for these outcomes as a function of transformed bio-ADM (pg/mL) using the natural logarithm.

Fitting logistic regression models adjusted for either sex and age or sex, age, NT-proBNP, CRP, and serum creatinine, for 90-day mortality using a continuous and standardized scale of bio-ADM levels (‘per IQR from the median of log-transformed bio-ADM’) yielded a more than doubling of the OR using the simpler model adjusted for sex and age only (*p* < 0.001), while it corresponded to a 50% increase (*p* = 0.002) in OR in the fully adjusted model. Using bio-ADM quartiles as an ordinal variable as a predictor of 90-day mortality and comparing the fourth quartile with the first, we observed an OR corresponding to four times increase (*p* < 0.001) in the model adjusted for sex and age, and a doubling of the OR in the fully adjusted model (*p* < 0.05). Details of the logistic regression models for 90-day mortality are summarized in Table 2.

In addition, we analyzed bio-ADM prediction for both 7 and 30-day mortality. 29 patients (2%) died within 7 days while 86 patients (6%) died within 30 days of ED presentation. Logistic regression analysis adjusted for sex and age with bio-ADM as a standardized continuous predictor yielded an OR of 2.7 (95% CI 1.8−4.2, *p* < 0.001) and 2.1 (95% CI 1.6−2.8, *p* < 0.001) for 7 and 30-day mortality respectively. Bio-ADM prediction remained statistically significant for 30-day mortality [OR 1.5 (95% CI 1.0−2.0, *p* = 0.03)] after additional adjustment for other biomarkers (CRP, creatinine, NT-proBNP), but not for 7-day mortality [OR 1.7 (95% CI 1.0−2.9, *p* = 0.06)]. See supplementary Table S1 for details.

Additional sub-analyses were performed to evaluate bio-ADM mortality prediction among hospitalized and discharged patients, and among patients 65 years or older versus patients younger than 65. 90-day mortality among discharged patients was 2.9% (17 out of 580), while it was 19.7% among admitted patients (162 out of 822). Logistic regression model adjusted for sex and age yielded bio-ADM OR 1.6 (95% CI 0.8−3.1, *p* = 0.175) among discharged and bio-ADM OR 1.8 (95% CI 1.5−2.3, *p* < 0.001) among admitted patients respectively. Moreover, 90-day mortality was 4.6% (18 out of 396) and 16% (161 out of 1006) in patients younger than 65 and patients 65 or older respectively. In models adjusted for sex, bio-ADM OR was 2.2 (95% CI 1.3−3.6, *p* = 0.002) among patients younger than 65, and bio-ADM OR 2.2 (95% CI 1.7−2.3, *p* < 0.001) for patients 65 years or older. For a complete summary, see Supplementary Table S2.

Further exploration of the study subjects showed that 8 (0.6%) patients were diagnosed with pneumothorax on either chest X-ray or computed tomography scans of the thorax. Excluding these patients did not statistically affect bio-ADM mortality prediction. See Supplementary Table S3.

### Secondary outcomes—hospitalization, IV diuretic therapy, heart-failure diagnosis at discharge

In total 822 (59%) patients were admitted to the hospital. We observed a higher proportion of hospitalization among patients with elevated bio-ADM levels. The proportion increases per quartile in ascending order where 35%, 50%, 70% and 81% of patients were admitted for in-hospital work-up or treatment. Fitting a logistic regression model for hospital admission adjusted for sex and age or sex, age, NT-proBNP, serum creatinine, and CRP with bio-ADM as a predictor on a continuous standardized scale we see a doubling of the OR (*p* < 0.001) in the simpler model (sex and age-adjusted) and a 50% increase of the OR in the fully adjusted model (*p* < 0.001). In quartile analysis of bio-ADM prediction for hospitalization, comparing the fourth quartile to the first yielded a five-fold increase of the OR compared to the first quartile (*p* < 0.001) in the sex and age-adjusted model, while this estimate was almost corresponding to a three-fold increase of the OR for the fourth versus first quartile in the fully adjusted model (*p* < 0.001). (See Supplementary Table S4).

In the study population, we observed an association with the use of intravenous (IV) diuretic treatment at the ED and increasing bio-ADM levels as well as CHF diagnosis and increasing bio-ADM levels. Overall 384 (28.1%) patients received IV diuretics while 262 (19%) patients had CHF as a diagnosis in their MHR upon discharge from an in-hospital ward or ED. Half of the patients receiving IV diuretics and more than a third who had CHF as a diagnosis at discharge belonged to the top quartile of bio-ADM concentrations (IV diuretics: 6%, 21%, 35%, and 50% and CHF at discharge: 4%, 11%, 24% and 35% of each bio-ADM quartile in ascending order).

Fitting logistic regression models adjusted for sex and age, with bio-ADM (continuous standardized scale) as a predictor for either treatment with IV diuretics or CHF as a diagnosis at discharge from hospital, yielded ORs of 2.6 (95% CI 2.1−3.1, *p* < 0.001) for IV treatment with diuretics and 2.5 (95% CI 2.1−3.1, *p* < 0.001) for predicting CHF as a diagnosis at discharge. On the contrary, bio-ADM was inversely associated with COPD [OR: 0.8 (95% CI 0.6−0.9, *p* = 0.012)] and was not associated with severe infection, like pneumonia, at discharge [OR: 1.0 (95% CI 0.8−1.3, *p* = 0.9)]. Model details are summarized in Supplementary Table S5.

### Discriminatory characteristics of bio-ADM—receiver operating characteristics

**Fig. 4:**
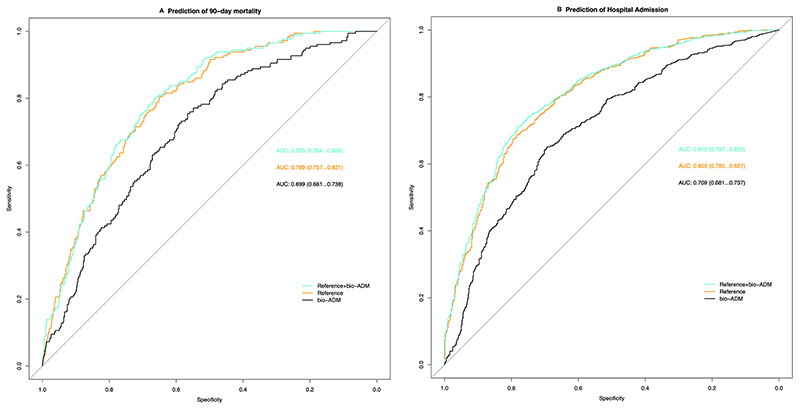
Receiver operating characteristics curve comparison between reference model, bioactive adrenomedullin (bio-ADM) and a combined model

To assess the overall capacity of bio-ADM to discriminate for 90-day mortality we computed the area under the curve (AUC) for bio-ADM alone, a model based on age, sex, NT-proBNP, CRP and serum creatinine (reference model), and a third model with bio-ADM added on top of the reference model (Fig. 3). The AUC for 90-day mortality of the reference model was 0.79 (95% CI 0.76−0.82) while adding bio-ADM on top of that model yielded an improved AUC of 0.80 (95% CI 0.76−0.83, added *χ*^2^ 9.8, *p* = 0.002). Computing AUCs for bio-ADM prediction of hospitalization, the reference model AUC was 0.80 (95% CI 0.78−0.83) while that of the full model including bio-ADM improved to 0.81 (95%CI 0.79−0.83, added *χ*^2^ 14.1, *p* = 0.0002). (Fig. 4).

In addition, to further explore the discriminatory capacity of bio-ADM we determined the optimal cutoff threshold for CHF diagnosis at discharge from the hospital. Using Youden’s *J* statistic, the specified threshold was ~ 29 pg/mL and an AUC 0.73 (95% CI 0.69−0.76). See Supplementary Figure S2.

Similarly, optimizing the cutoff threshold for 90-day mortality yields a threshold value corresponding to 29 pg/mL (75% sensitivity, 56.5% specificity). Fitting a logistic regression model for 90-day mortality adjusted for sex and age using the bio-ADM cutoff 29 pg/mL as a binary variable yields an OR 2.9 (95% CI 2.0−4.2, *p* < 0.001). Survival curves for 90-day mortality stratified for 29 pg/mL are shown in Supplementary Figure S3.

## Discussion

In this prospective observational study of 1402 unselected patients who sought urgent care due to dyspnea, the key finding is that a single measurement of bio-ADM predicts 90-day mortality independently of traditional prognostic biomarkers.

In addition, elevated concentrations of bio-ADM are associated with heart failure and decongestive treatment, being independently associated with hospitalization with the need for IV diuretic treatment and CHF diagnosis at discharge.

To our knowledge, this is the first time bio-ADM has been studied in an unselected dyspneic population at the ED, where immediate care and management are dictated by symptoms rather than diagnoses. Our results are in line with recent studies [17–21, 27], all of which differ in the sense that they were performed in cohorts selected for CHF of different acuity and severity. These studies all found bio-ADM to be a valuable addition to our clinical toolkit as a biomarker of congestion.

In our cohort, median bio-ADM concentration was 27.9 (IQR: 16.9−49.6) pg/mL, while the median concentration in a healthy population has been reported to be 20.7 pg/mL (99th percentile: 43 pg/mL) [15] and recent studies on CHF patients report median values between 33.8 (IQR: 22.6−53.9) [16] and 44.1 (IQR: 25.9−82.7) [17] pg/mL. Further, it is discussed that the bio-ADM level seems to reflect both the acuity and the severity of CHF-related fluid overload [21].

Our results show that bio-ADM predicts 90 day all-cause mortality independently of several other known risk factors including NT-proBNP. Also, in comparison of logistic regression models for 90 day mortality and hospitalization, we see that the addition of bio-ADM on top of a reference model containing age, sex and established biomarkers CRP, creatinine and NT-proBNP is statistically significant. Interestingly, Ter Maaten and colleagues [19] found bio-ADM was a weaker predictor for mortality prediction in a CHF population compared to NT-proBNP. This is concordant with our results of the general dyspneic patient group where we see a higher point-estimate for 90 day mortality of NT-proBNP compared to bio-ADM.

Further, we show that a single measurement of bio-ADM in dyspneic patients at ED presentation is associated with hospitalization, subsequent IV diuretic treatment at the ED and a CHF diagnosis at discharge, which altogether points toward congestive problems underlying the experienced shortness of breath. These findings support the notion that endothelial dysfunction represented by increased bio-ADM is an effective biomarker of (extravascular) congestion in patients with undifferentiated dyspnea, and that it may add clinically important information on the need of early decongestive therapy at the ED.

This is in line with previous studies on acute heart failure, showing an independent association between bio-ADM concentration and severe congestion [18, 20]. Interestingly, through repeated measurements of bio-ADM, Kremer and colleagues [20] found that bio-ADM decreases with appropriate decongestion and symptom relief over a 7 day period, in contrast to BNP where they could not identify this response. In addition, it has been shown that bio-ADM levels reflecting more severe and widespread congestion correlate positively with a patient’s standing dose of maintenance diuretics [19, 21].

In a recent publication [21] bio-ADM was evaluated at discharge in CHF patients. The authors showed that higher bio-ADM levels at discharge are associated with a longer hospital stay, worse response to diuretic treatment and hospital readmission, further reinforcing bio-ADM as a potential therapy guiding biomarker.

In the longer term, bio-ADM is of particular interest as a drug target. Adrecizumab (Adrenomed AG, Hennigsdorf, Germany) is a monoclonal antibody binding the N-terminus of bio-ADM which leads to dose-dependent increase in plasma bio-ADM, through a ‘trapping’ mechanism, preventing the bio-ADM molecules from extravasation due to its high molecular weight (160 kDa). Yet, the receptor binding C-terminal part of bio-ADM is left free, leading to increased stimulation of endothelial ADM receptors with resulting improvement of vascular integrity and reduced edema and congestion [2]. The clinical effects of Adrecizumab are being tested both in human subjects with septic shock (AdrenOSS-2, NCT03085758) and in acute heart failure [2]. Depending on the outcome of these trials, patients with high bio-ADM indicating ongoing endothelial dysfunction might turn benefit from further decongestive therapy with Adrecizumab through enhanced bio-ADM effects on the endothelium leading to improved vascular integrity and less fluid extravasation.

### Strengths and limitations

We believe the diverse and large study population of dyspneic patients we have studied is a realistic representation of a true ED setting which is a significant strength of this study. Inclusion and comparison with an established biomarker of heart failure such as natriuretic peptide in our assessment of bio-ADM is another strength.

Some limitations of our study are intrinsic to the study design. Being a single center prospective observational study, naturally causality cannot be shown and extrapolation to other ED populations cannot directly be done. Also, it is important to note that the inclusion of patients in the study was done solely during office hours and required the patient to be in condition to give consent and answer questions at the ED. This may have introduced selection bias towards patients with an overall better general condition. Also, patients were not prospectively followed-up but mortality rate (primary endpoint) was determined by using a National Registry. The absence of data on and lack of comparison to other diagnostic tools available to emergency physicians at the bedside, such as lung and heart ultrasound can be viewed as a limitation.

## Conclusion

We show for the first time that high plasma bio-ADM measured during the first hour at the ED in unselected acutely dyspneic patients is independently associated with 90 day mortality and need of hospitalization. Our data suggest that early decongestion therapy might be of value in patients with endothelial dysfunction according to high bio-ADM, which is supported by the statistically significant improvement in model fit (ie added *χ*^2^) for both 90-day all-cause mortality and hospitalization prediction after the addition of bio-ADM and association of bio-ADM with IV diuretic treatment. Whether such therapy improves prognosis in acutely dyspnoeic patients with high bio-ADM should be addressed in future biomarker-guided intervention studies.

## Supplementary Material

Fig 1-3

Table 1-5

## Figures and Tables

**Table 1:** Study population characteristics (*n* = 1402)

	Bio-ADM quartiles				All patients	*P* for trend
	Quartile 1 (*N* = 351)	Quartile 2 (*N* = 350)	Quartile 3 (*N* = 351)	Quartile 4 (*N* = 350)	Overall (*N* = 1402)	
Bio-ADM (pg/mL)^a^						
Median	12	22	36	78	28	
Range	8−17	17−28	28−50	50−995	8−995
Age, mean (SD)	62.3 (19.0)	70.7 (17.8)	75.8 (13.3)	75.5 (15.3)	71.1 (17.4)	**
Female sex	210 (59.8%)	204 (58.3%)	185 (52.7%)	180 (51.4%)	779 (55.6%)	**
CHF^b^	41 (11.7%)	78 (22.3%)	154 (43.9%)	208 (59.4%)	481 (34.3%)	***
COPD^c^	94 (26.8%)	103 (29.4%)	127 (36.2%)	112 (32.0%)	436 (31.1%)	*
Infection^d^	80 (22.8%)	88 (25.1%)	118 (33.6%)	107 (30.6%)	393 (28.0%)	0.99
Missing	1 (0.3%)	2 (0.6%)	1 (0.3%)	3 (0.9%)	7 (0.5%)	
Biomarker measurements						
CRP^e^ (mg/L)						**
Median	4	7	16	16	10	
IQR	1−14	3−27	5−49	6−46	3−35	
NT-proBNP^f^ (pg/mL)						**
Median	133	481	1860	4180	869	
IQR	54−494	127−2243	436−4508	1235−10,292	153−3828	
Creatinine (μmol/L)						**
Median	69	76	91	100	80	
IQR	57−81	65−89	71−121	76−148	65−106	

a Bioactive adrenomedullin

b Congestive heart failure in past medical history

c Chronic obstructive pulmonary disease in past medical history

d Undifferentiated infection as per triage at emergency department

e C-reactive protein

f N-terminal pro-B-type natriuretic peptide

* *p* < 0.05

** *p* < 0.005

*** *p* < 0.001

**Table 2:** Logistic Regression model summary with respect to 90 day mortality according to bio-ADM quartiles

	All patient (*n* = 1402) [8−995]	*P* value	Quartile 1 (n = 351) [8−17]	Quartile 2 (*n* = 350) [17−28]	Quartile 3 (*n* = 351) [28−50]	Quartile 4 (*n* = 350) [50−995]
90 day mortality						
*N* events (% of total)	179 (12.8%)		15 (4.3%)	26 (7.4%)	57 (16.2%)	81 (23.1%)
Unadjusted OR (95% CI)^b^	2.1 (1.7−2.6)	< 0.001	Reference	1.3 (0.7−2.6)	2.70 (1.51−5.10)^**^	4.2 (2.4−7.9)^***^
Adjusted OR (95% CI)^c^	1.5 (1.2−2.0)	0.002	Reference	1.0 (0.5−2.1)	1.6 (0.9−3.3)	2.1 (1.1−4.3)^*^

Odds Ratio (OR) concerning all patients is based on a continuous scale of log-transformed biomarker-values centered around the median and defined as ‘per interquartile range (IQR) from median of log-transformed bio-ADM’. Other biomarkers included in the model were also log-transformed and centered around the median in the same fashion. Adjusted ORs displayed in quartile columns are based on the first quartile of the other biomarkers included in the model [C-reactive protein (CRP), serum creatinine, N-terminal pro-B-type natriuretic peptide (NT-proBNP)]

a Bioactive adrenomedullin measured in plasma

b ORs for logistic regression model adjusted for sex and age

c ORs for logistic regression model also adjusted for CRP, serum creatinine, NT-proBNP in addition to sex and age

* *p* < 0.05

** *p* < 0.01

*** *p* < 0.001

## Data Availability

The datasets generated during and/or analysed during the current study are available from the corresponding author on reasonable request.
