# Supplementary material for: Bioactive adrenomedullin a prognostic biomarker in patients with mild to moderate dyspnea at the emergency department: an observational study
Source: Intern Emerg Med. Author manuscript; Available in PMC 2022 May 26. (PMC8964625; doi:10.1007/s11739-021-02776-y)

Supplementary Information

11739_2021_2776_MOESM1_ESM.tiff

Supplementary Figure S1–Plots illustrating the change in odds as a function of transformed bioADM using the natural logarithm. Illustrations of change in Log Odds for (A) 90-day mortality and (B) hospital admission as a function of transformed bioADM (pg/mL) using the natural logarithm (TIFF 1165 kb)

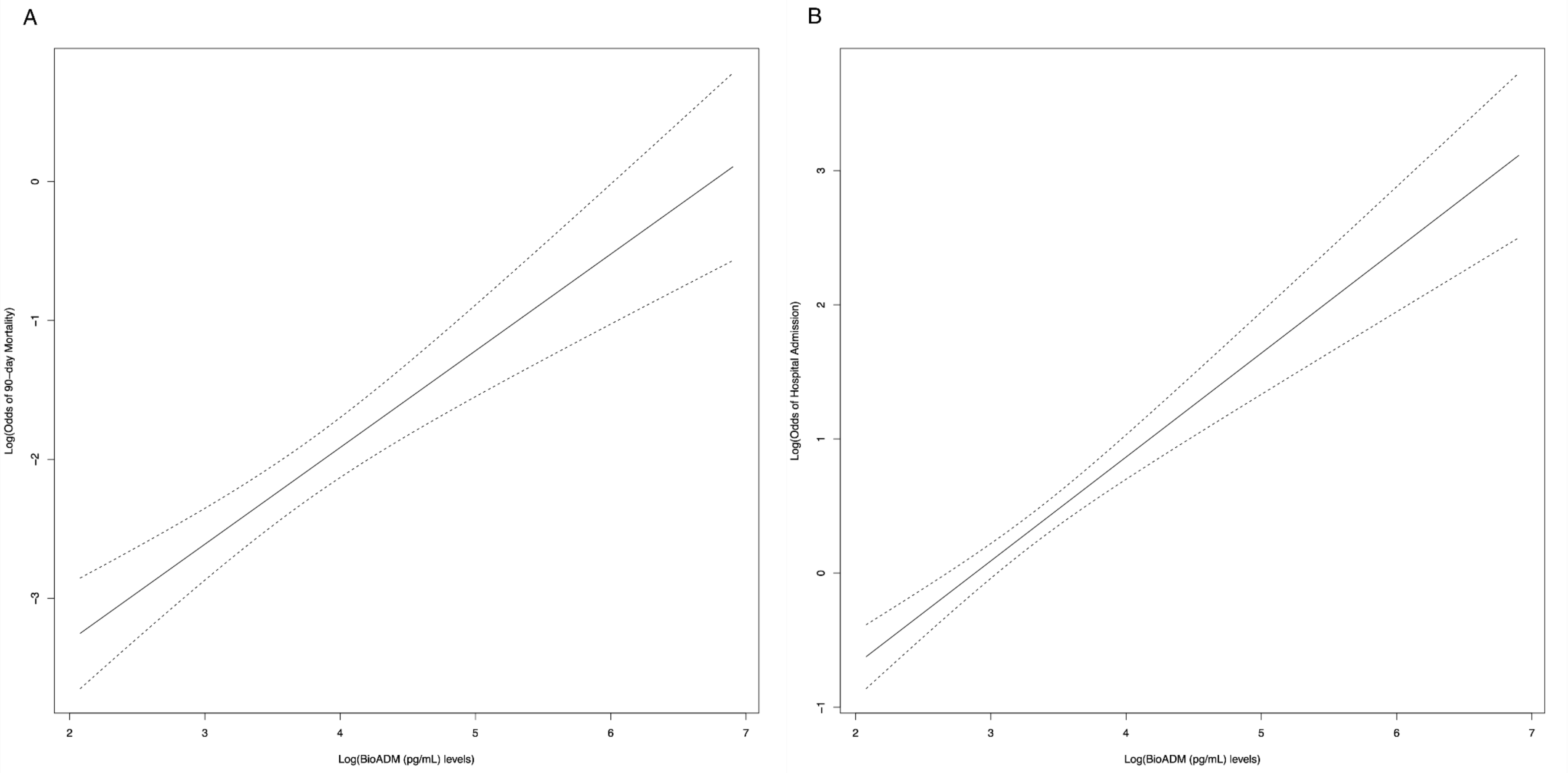

11739_2021_2776_MOESM2_ESM.tiff

Supplementary Figure S3–Survival curves for 90-days stratified for bioADM cutoff 29pg/mL. Note cut x-axis at 0.75 (TIFF 50628 kb)

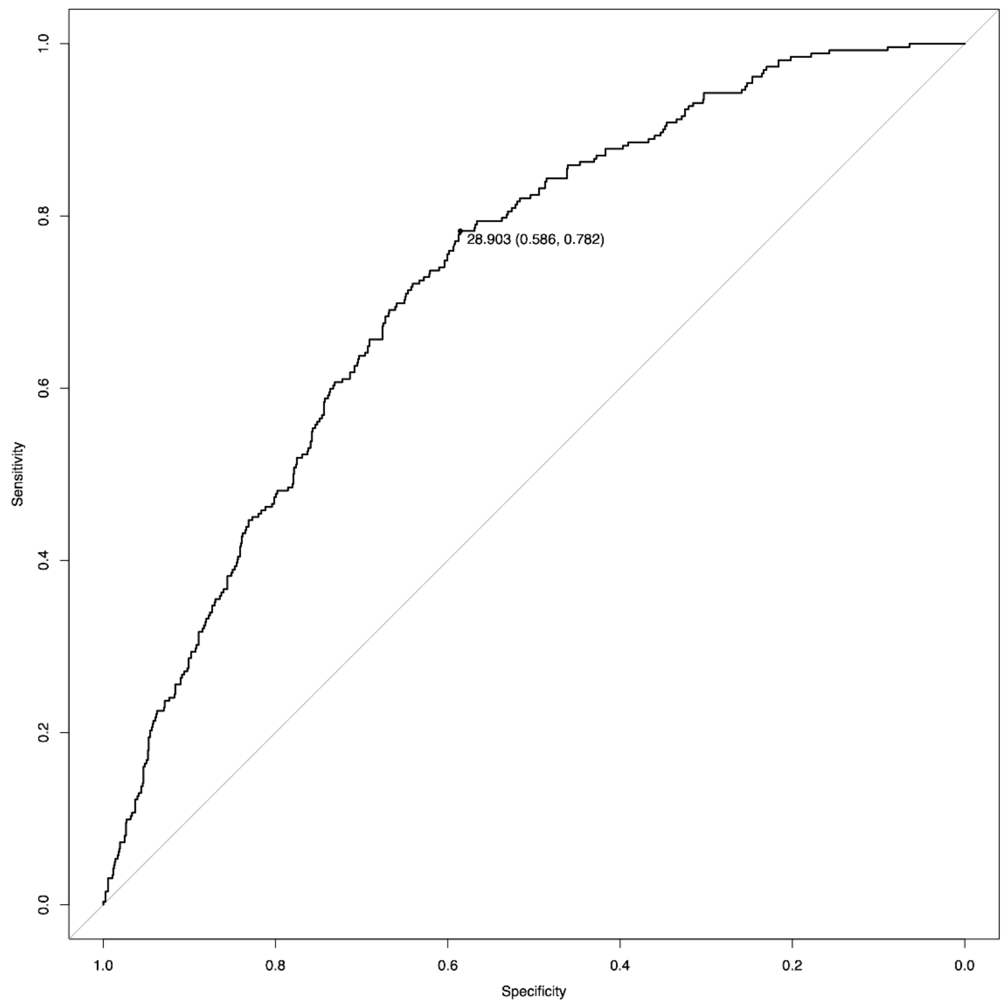

11739_2021_2776_MOESM3_ESM.tiff

Supplementary Figure S2–ROC-plot for bioADM discrimination for CHF diagnosis at discharge from ED or ward. Suggested bioADM cutoff: 28.9pg/mL; AUC 0.73 (95% CI: 0.69-0.76). (TIFF 50628 kb)

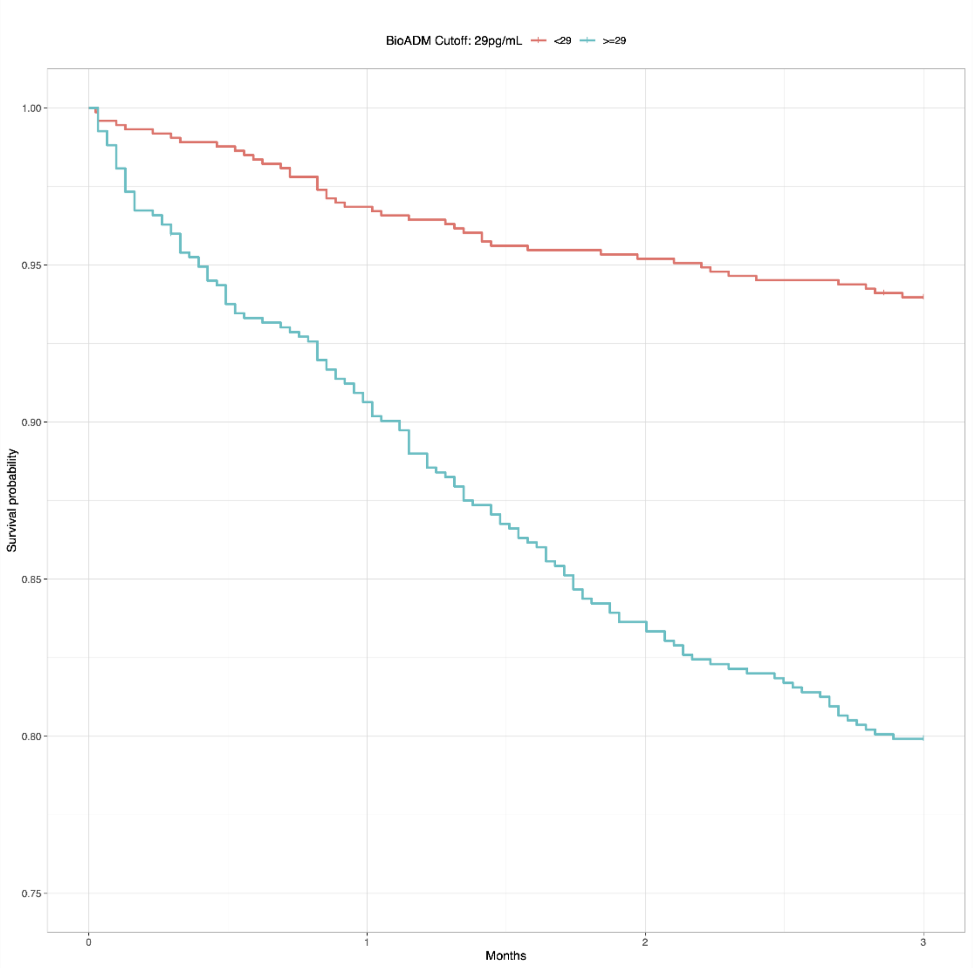

Supplement: Fig 1-3 [file EMS143774-supplement-Fig_1_3.pdf]
