# Supplementary material for: Bioactive adrenomedullin a prognostic biomarker in patients with mild to moderate dyspnea at the emergency department: an observational study
Source: Intern Emerg Med. Author manuscript; Available in PMC 2022 May 26. (PMC8964625; doi:10.1007/s11739-021-02776-y)
Supplement: Table 1-5 [file EMS143774-supplement-Table_1_5.pdf]

Supplementary Table S1 – Logistic regression model for 7- & 30-day mortality prediction (n=1402).

Mortality	7-day			30-day		
**Predictors**		**95% CI**	**p**		**95% CI**	**p**
**Unadjusted OR**[a]	2.7	1.8 – 4.2	<0.001	2.1	1.6 – 2.8	<0.001
**Adjusted OR**[b]	1.7	1.0 - 2.9	0.058	1.5	1.0 - 2.0	0.030

[a]Bioactive adrenomedullin, measured as per increase of one IQR from Median of log-transformed bio-ADM (pg/mL); Odds Ratio (OR) adjusted for sex and age.
[b]ORs for logistic regression model also adjusted for standardized log transformed biomarkers CRP, serum creatinine, NT-proBNP in addition to sex and age.

Supplementary Table S2 – Mortality prediction study sub-populations based on age and hospital admission status.

**Mortality**	**90 days**			**30 days**			**7 days**		
	OR	95% CI	p	OR	95% CI	p	OR	95% CI	p
**Discharged**[a] (*n*=580)	1.6	0.8 – 3.1	0.175	1.2	0.2 – 5.0	0.81	0.2	0.00 – 6.0	0.522
**Admitted**[a] (*n*=822)	1.8	1.5 – 2.3	**<0.001**	1.8	1.3 – 2.4	**<0.001**	2.5	1.6 – 4.0	**<0.001**
**≥65 years**[b] (*n*=1006)	2.2	1.7-2.7	**<0.001**	2.1	1.6-2.8	**<0.001**	3.0	1.9-4.8	**<0.001**
**<65 years**[b] (*n*=396)	2.2	1.3-3.7	**0.002**	2.5	1.2-5.0	**0.011**	1.5	0.3-4.4	**0.52**

Odds ratios express bioactive adrenomedullin, measured as per increase of one IQR from Median of log-transformed bio-ADM (pg/mL)
[a]Model adjusted for sex and age.
[b]Model adjusted for sex.

Supplementary Table S3 – Logistic regression of 90-day mortality prediction after exclusion of patient with radiological evidence of pneumothorax (*n*=1394).

	**90-day mortality**		
**Model**	OR	95% CI	p
**Unadjusted OR**[a]	2.1	1.7 – 2.6	**<0.001**
**Adjusted OR**[b]	1.5	1.2 – 2.0	**0.002**

Odds Ratio expressed as Bioactive adrenomedullin, measured as per increase of one IQR from Median of log-transformed bio-ADM (pg/mL)
[a]Model Odds Ratio (OR) adjusted for sex and age.
[b]ORs for logistic regression model also adjusted for standardized log transformed biomarkers CRP, serum creatinine, NT-proBNP in addition to sex and age.

Supplementary Table S4. Logistic regression analysis of bioADM prediction for hospital admission.

**Hospital admission**						
**bioADM Quartile**			**1**	**2**	**3**	**4**
**N events (% of total)**	822 (58.6%)		121 (34.5%)	174 (49.6%)	244 (69.5%)	283 (80.9%)
**Unadjusted OR (95% CI)[b]**	2.3 (1.9-2.7)	**<0.001**	Reference	1.4 (1.0-2.0)*	2.8 (2.0 – 4.0)***	5.6 (3.9 – 8.2)***
**Adjusted OR (95% CI)[c]**	1.5 (1.2-1.8)	**<0.001**	Reference	1.1 (0.8-1.6)	1.6 (1.1-2.4)*	2.8 (1.8-4.3)***

Odds Ratio (OR) concerning all patients is based on a continuous scale of log-transformed biomarker-values centered around the median and defined as 'per interquartile range (IQR) from median of log-transformed bio-ADM'. Other biomarkers included in the model were also log-transformed and centered around the median in the same fashion. Adjusted ORs displayed in quartile columns are based on the first quartile of the other biomarkers included in the model (C-reactive protein [CRP], serum creatinine, N-terminal pro-B-type natriuretic peptide [NT-proBNP]).

[a]bioactive adrenomedullin measured in plasma

[b]ORs for logistic regression model adjusted for sex and age. [c]ORs for logistic regression model also adjusted for CRP, serum creatinine, NT-proBNP in addition to sex and age.

*p<0.05, **p<0.01, ***p<0.001

Supplementary Table S5. Logistic Regression model adjusted for sex and age with respect to association of bio-ADM with intravenous diuretic treatment & CHF diagnosis at discharge (n=1402).

	**IV diuretics**			**CHF[b] at discharge**		
**Predictors**	OR[c]	95% CI[d]	p	OR	95% CI	p
**Bio-ADM[a]**	2.6	2.1 – 3.1	**<0.001**	2.5	2.1 – 3.1	**<0.001**
**Male Sex**	1.6	1.2 – 2.0	**0.001**	1.83	1.4 – 2.5	**<0.001**
**Age**	1.1	1.0 – 1.1	**<0.001**	1.1	1.0 – 1.1	**<0.001**

[a]Bioactive adrenomedullin, measured as per increase of one IQR from Median of log-transformed bio-ADM (pg/mL).
[b]Congestive heart failure. [c]Odds ratio. [d]Confidence interval
